# Immune checkpoint inhibitors-associated thrombosis in patients with head and neck cancer: a study of the Spanish society of medical oncology (SEOM) thrombosis and cancer group

**DOI:** 10.1007/s12094-024-03570-w

**Published:** 2024-06-21

**Authors:** Manuel Sánchez Cánovas, Miguel Ángel Moya Hernández, Evdochia Adoamnei, Diego Cacho Lavin, David Fernández Garay, Teresa Quintanar Verdúguez, Jacobo Rogado Revuelta, Francisco José García Verdejo, Silvia García Adrián, Ana Isabel Ferrer Pérez, María Esperanza Guirao García, Javier López Robles, Jaime Mendiola, Andrés J. Muñoz Martín

**Affiliations:** 1 Spanish Society of Medical Oncology (SEOM) Thrombosis and Cancer Group, Madrid, Spain; 2 Medical Oncology Department, Hospital Universitario Morales Meseguer, Murcia, Spain; 3 Nursing Department, Nursing Faculty, University of Murcia, IMIB-Arrixaca, Murcia, Spain; 4 Medical Oncology Department, Hospital Universitario Marqués de Valdecilla, Santander, Spain; 5 Medical Oncology Department, Complejo Hospital Costa del Sol, Marbella, Spain; 6 Medical Oncology Department, Hospital General Universitario de Elche, Elche, Spain; 7 Medical Oncology Department, Hospital Universitario Infanta Leonor, Madrid, Spain; 8 Medical Oncology Department, Complejo Hospitalario de Jaén, Jaén, Spain; 9 Medical Oncology Department, Hospital Universitario de Móstoles, Madrid, Spain; 10 Medical Oncology Department, Hospital Obispo Polanco, Teruel, Spain; 11 School of Medicine, Social and Health Sciences, University of Murcia, IMIB-Arrixaca, Cyber Epidemiology and Public Health (CIBERESP), Murcia, Spain; 12 Medical Oncology Department, Hospital General Universitario Gregorio Marañón, Madrid, Spain

**Keywords:** Immune Checkpoint Inhibitors, Cancer-related thrombosis, Head and neck cancer

## Abstract

**Purpose:**

Both venous and arterial thrombotic events (VTE/AT) can be associated with Immune Checkpoint Inhibitors (ICI). However, there is a paucity of information apropos patients in routine clinical practice.

**Methods:**

/Patients.

This retrospective, multicenter study was promoted by the Thrombosis and Cancer Section of the Spanish Society of Medical Oncology (SEOM). Individuals with head and neck cancer who initiated ICI between 01/01/2015 and 31/12/2021 were recruited. Minimum follow-up was 6 months (except in cases of demise).

The primary objective was to calculate the incidence of ICI-associated VTE/AT, with secondary objectives including the analysis of their impact on survival and the identification of variables predictive of VTE/AT.

**Results:**

A total of 143 patients with head and neck cancer were enrolled. The incidence of VTE/AT during follow-up (median 8.6 months) was 2.8%. Survival analysis showed no significant differences (p = 0.644) between the group that developed VTE/AT (median 7.13 months, 95% CI 0–22.9) and the group that did not (median 9.86 months, 95% CI 6.3–13.4). The presence of liver metastases was predictive of VTE/AT (p < 0.05).

**Conclusions:**

Thromboembolic disease associated with immunotherapy in patients with head and neck neoplasia does not significantly impact survival. The presence of liver metastases can predict these events.

## Introduction

Immune Check Point Inhibitors (ICI) have emerged as pivotal agents in the management of oncologic patients in recent years. This paradigm shift has prompted investigations utilizing real-world clinical data to complement findings from randomized clinical trials (RCTs), aiming to enhance our understanding of efficacy and elucidate unregistered toxicities. Among the spectrum of adverse events associated with ICI therapy, venous thrombotic events (VTE) and arterial thrombotic events (AT) have garnered significant attention.

The Cancer-Associated Thrombosis Group, affiliated with the Spanish Society of Medical Oncology (SEOM), has contributed two seminal studies addressing these concerns. The initial investigation focused on patients afflicted with melanoma and lung carcinoma, revealing a substantial impact of thrombotic events on overall survival. Moreover, biomarkers such as the neutrophil-to-lymphocyte ratio and lactate dehydrogenase emerged as potential predictors of thrombotic complications [1]

Subsequently, a follow-up study examined cohorts comprising renal and bladder neoplasms. While survival outcomes remained unaffected, a noteworthy association between serum albumin levels and thrombotic risk in bladder cancer patients receiving ICI therapy was identified [2].

Collectively, these findings underscore the imperative of continued inquiry into thrombotic phenomena across diverse oncologic cohorts. Herein, we present our investigation elucidating thrombotic events in patients diagnosed with head and neck tumors (HNC).

## Material and methods

This study has been sponsored by the SEOM Thrombosis and Cancer Section. It is a retrospective, multicenter study (9 centers). Data from patients with head and neck cancer who initiated ICI between 01/01/2015 and 31/12/2021 were collected. Selection was independent of tumor stage, type of ICI, or treatment intent. Participants had to have a minimum follow-up of 6 months (unless this was impossible due to patient demise).

The primary objective was to calculate the incidence of thrombosis associated with ICI. Two secondary objectives were defined. The first was to examine the impact of thrombosis on survival among subjects treated with ICI, while the second was to find predictor variables for the development of VTE/TA.

Median and interquartile range (IQR) 25–75 were used to describe quantitative characteristics. Qualitative characteristics were reported by number (n) and percentage (%). Survival analysis was performed using the Kaplan–Meier estimator and log-rank test, calculating the median and 95% confidence intervals (CI) of survival times. In addition, analyses were performed with the “Landmark" method at 3, 6, and 9 months of follow-up from the time ICI therapy was initiated. To determine predictor variables, multivariate logistic regression models were performed to obtain Odds Ratios (OR) and 95% CI. Statistical significance was set at a p-value of 0.05 and the SPSS 25.0 statistical package (IBM Corporation, Armonk, NY, USA) was used.

The study was submitted to the Ethics Committee of each participating center and obtained the corresponding approval prior to its commencement. The processing, communication, and transfer of all personal data complied with the provisions of Organic Law 15/1999, dated December 13, 1999, regarding the protection of personal data and of Organic Law 3/2018, dated December 5, 2018, since it came into force.

## Results

A total of 143 patients were recruited, and baseline characteristics are presented in Table 1. The cohort was predominantly male (87.4%) with a median age of 63 years (interquartile range [IQR] 56–70). Functional status was predominantly good, with 70% of patients having an Eastern Cooperative Oncology Group (ECOG) performance status of 0–1, although nearly 30% had an ECOG of 2. Squamous cell carcinoma was the predominant histology (96.5%), and the majority of patients (93%) presented with disseminated oncological disease (stage IV) at the initiation of ICI.

**Table 1:** Baseline characteristics of head and neck cancer patients (complete population and cohort with VTE/ AT associated with ICI)

Parameter	Subparameter	Complete population (n = 143)	Cohort with VTE/ AT (n = 4)
Gender	Male	87.4% (n = 125)	50% (n = 2)
	Female	12.6% (n = 18)	50% (n = 2)
BMI	< 18.5 kg/m2	9.7% (n = 14)	0% (n = 0)
	18.5 – 24.9 kg/m2	49.9% (n = 70)	25% (n = 1)
	25 – 29.9 kg/m2	30.7% (n = 44)	50% (n = 2)
	> 30 kg/m2	6.2% (n = 9)	25% (n = 1)
	Not available	3.5% (n = 6)	0% (n = 0)
Smoking status	Never smoked	10.5% (n = 15)	25% (n = 1)
	Active smoker	39.9% (n = 57)	25% (n = 1)
	Ex-smoker	49.6% (n = 71)	50% (n = 2)
Medical history unrelated to the current head and neck cancer	HTA	42% (n = 60)	50% (n = 2)
	DM	21% (n = 30)	75% (n = 3)
	DLP	35.7% (n = 51)	50% (n = 2)
	Thrombophilia	0.7% (n = 1)	0% (n = 0)
	History of cardiovascular pathology (AMI…)	18% (n = 26)	25% (n = 1)
	COPD	19.6% (n = 28)	25% (n = 1)
	Autoimmune disease	2.1% (n = 3)	0% (n = 0)
	Liver disease	15.4% (n = 22)	25% (n = 1)
	CKD	6.4% (n = 9)	0% (n = 0)
	CVD	0.7% (n = 1)	0% (n = 0)
	Other previous malignancies	9.9% (n = 14)	0% (n = 0)
	VTE/ AT (Diagnosed at least 30 days prior to the detection of head and neck cancer)	1.4% (n = 2)	0% (n = 0)
	VTE/ AT (Diagnosed between cancer diagnosis and ICI initiation)	8.4% (n = 12)	0% (n = 0)
	Concomitant hormonal therapy	0% (n = 0)	0% (n = 0)
	Concomitant EPO	0.7% (n = 1)	0% (n = 0)
	PICC or port-a-cath carrier	26.6% (n = 38)	50% (n = 2)
Tumor stage at ICI initiation	Stage III	7% (n = 10)	0% (n = 0)
	Stage IV	93% (n = 133)	100% (n = 4)
Histology	Epidermoid	96.5% (n = 138)	100% (n = 4)
	Non epidermoid	3.5% (n = 5)	0% (n = 0)
Location	Nasopharynx	7.7% (n = 11)	25% (n = 1)
	Oropharynx	41.3% (n = 59)	25% (n = 1)
	Hypopharynx	15.4% (n = 22)	25% (n = 1)
	Larynx	25.2% (n = 36)	0% (n = 0)
	Oral cavity	5.5% (n = 8)	25% (n = 1)
	Maxillary sinus	1.4% (n = 2)	0% (n = 0)
	Parotid	0.7% (n = 1)	0% (n = 0)
	Unknow origin	2.8% (n = 4)	0% (n = 0)
P16/HPV	Negative	47.6% (n = 68)	25% (n = 1)
	Positive	9.8% (n = 14)	0% (n = 0)
	Unknow	42.6% (n = 61)	75% (n = 3)
PDL-1	Negative	7% (n = 10)	0% (n = 0)
	Positive	10.5% (n = 15)	25% (n = 1)
	Unknow	82.5% (n = 118)	75% (n = 3)
Involvement of the cervical vascular bundles	No	86.7% (n = 124)	75% (n = 3)
	Yes	13.3% (n = 19)	25% (n = 1)
Stage IV	Liver metastases	7.7% (n = 11)	50% (n = 2)
	Lung metastases	43.4% (n = 62)	75% (n = 3)
	Central nervous system metastases	1.4% (n = 2)	0% (n = 0)
	Bone metastases	14.7% (n = 21)	25% (n = 1)
ECOG at start of ICI	0–1	70% (n = 100)	75% (n = 3)
	2	30% (n = 40)	25% (n = 1)
Treatment modality in which ICI was used	First-line metastatic disease	20.3% (n = 29)	0% (n = 0)
	Second-line metastatic disease	63.6% (n = 91)	100% (n = 4)
	Third or subsequent line of metastatic disease	14% (n = 20)	0% (n = 0)
	Adjuvant	2.1% (n = 3)	0% (n = 0)
Treatment regimen	Nivolumab in monotherapy	83.9% (n = 120)	100% (n = 4)
	Pembrolizumab in monotherapy	10.5% (n = 15)	0% (n = 0)
	Pembrolizumab + Chemotherapy	2.1% (n = 3)	0% (n = 0)
	Others	3.5% (n = 5)	0% (n = 0)
Status of last follow-up	Deceased	71.3% (n = 102)	100% (n = 4)
	Alive	28.7% (n = 41)	0% (n = 0)

*AMI* acute myocardial infarction, *AT* arterial thrombosis, *BMI* body mass index, *CKD* chronic kidney disease, *COPD* chronic obstructive pulmonary disease, *CVD* cerebrovascular disease, *DLP* dyslipemia, *DM* diabetes mellitus, *EPO* erythropoietin, *HPV* human papilloma virus, *HTA* arterial hypertension, *ICI* Immune Checkpoint Inhibitors, *PICC* peripherally inserted central catheter, *VTE* venous thromboembolism

ICI was primarily administered in the first line (20.3%) or second line (63.6%) for advanced disease. The majority of patients (84%) received nivolumab monotherapy as the chosen antineoplastic treatment modality.

With respect to thrombotic history, 1.4% of subjects had a documented history of VTE/AT, diagnosed at least 30 days prior to the detection of head and neck cancer. During the interval between cancer diagnosis and initiation of ICI, VTE or AT events occurred in 8.4% of cases.

The incidence of VTE/AT associated with ICI during the median follow-up period of 8.6 months was 2.8% (interquartile range [IQR]: 3.42–18.1) (n = 4). The baseline characteristics of patients experiencing VTE/AT episodes are summarized in Table 2.

**Table 2:** Characteristics of VTE/ AT episodes in patients with head and neck cancer

Parameter	Subparameter	n = 4
Type VTE/AT	PE	50% (n = 2)
	DVT	25% (n = 1)
	Other forms of VTE: visceral, associated with catheter…	25% (n = 1)
VTE/ AT presentation	Incidental	25% (n = 1)
	Symptomatic	75% (n = 3)
Setting of VTE/ AT diagnosis	Outpatient	75% (n = 3)
	In-patient	25% (n = 1)
Setting of VTE/ AT management	Outpatient	25% (n = 1)
	In-patient	75% (n = 3)

*AT* arterial thrombosis, *DVT* deep vein thrombosis, *PE* pulmonary embolism, *VTE* venous thromboembolism

At the time of VTE/AT diagnosis, patients had received a median of 2.5 ICI cycles (IQR 1.3–4.5), with all patients who experienced these complications having received nivolumab as second-line treatment. Additionally, 25% of patients with VTE/AT were receiving anticoagulant therapy (at prophylactic doses) at the time of the event. Pulmonary embolism (PE) was the most common form of thrombosis, accounting for 50% of cases.

Regarding other treatments potentially influencing thrombosis risk, 25% (n = 2) of patients were undergoing antibiotic treatment, and another 25% were receiving corticosteroid treatment. Furthermore, 25% of patients with thrombosis had undergone a surgical procedure in the previous month.

The majority of thrombotic events (75%) were symptomatic, with initial management primarily occurring in the hospital setting (75% of cases), despite most subjects (75%) being diagnosed in an outpatient setting. All patients received treatment with low molecular weight heparin, with 50% receiving treatment for more than 6 months. Following VTE/AT occurrence, ICI therapy was discontinued in 33.3% of patients. No instances of rethrombosis or bleeding events were observed during the post-VTE/AT follow-up period.

Multivariate analysis (Table 3) unveiled one variable exhibiting a statistically significant association with VTE/AT risk. This variable was the presence of liver metastases (HR 13.22; 95% CI 1.66–105.18).

**Table 3:** Multivariate analysis to detect the relationship between clinical variables and development of VTE/ AT in patients with head and neck cancer and ICI

	Multivariate analysis		
	HR	95% CI	p value
Liver metastases at initiation of ICI	13.22	1.66 – 105.18	0.01
Lung metastases at initiation of ICI	4.68	0.35 – 63.1	0.25
Bone metastases at initiation of ICI	0.26	0.01 – 17.22	0.56
ECOG at initiation of ICI (cutoff > 2)	0.10	0.00 – -	1.00
Hemoglobin at initiation of ICI (cutoff < 10 g/dl)	0.45	0.20- 1.25	0.23
Leukocytes at initiation ICI (cutoff < 10,000 cells/mm3)	0.40	0.02 – 6.87	0.52
Neutrophil/ lymphocyte ratio at initiation of ICI (cutoff < 3)	0.57	0.005 – 61.79	0.81
Platelet/ lymphocyte ratio at initiation of ICI (cutoff > 300)	1.54	0.07 – 34.85	0.79

*CI* confidence interval, *HR* hazard ratio, *ICI* immune checkpoint inhibitors

**Fig. 1:**
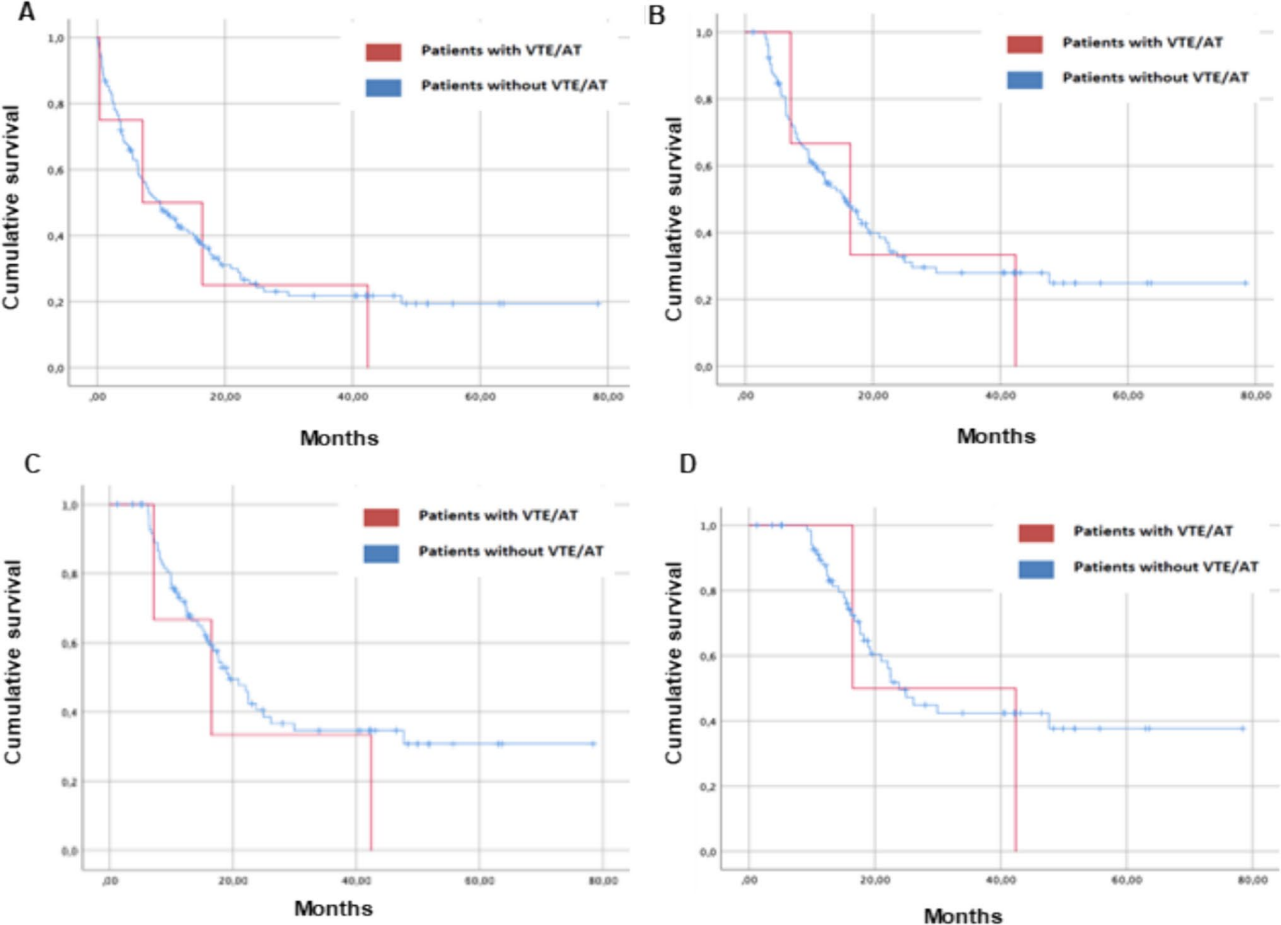
Survival analysis: **A** Kaplan Meier curve comparing OS (since initation ICI) of head and neck cancer patients treated with ICI who developed VTE/ AT versus those who did not; **B** Landmark analysis at 3 months after initation ICI; **C** Landmark analysis at 6 months after initation ICI; **D** Landmark analysis at 9 months after initation ICI

Survival analysis (Fig. 1A) revealed no significant differences (log-rank test = 0.64) between the group that developed VTE/TA (median 7.13 months, 95% CI 0 – 22.9) and those who did not (median 9.86 months, 95% CI 6.3 – 13.4).

Landmark analysis at 3 months (Fig. 1B) similarly demonstrated no significant differences in overall survival (median OS in VTE/AT group 16.46 months, 95% CI 1.53 – 31.40; non-VTE/AT group 15.64 months, 95% CI 11.27 – 20, p = 0.69).

Likewise, the 6-month landmark analysis (Fig. 1C) yielded consistent results indicating no significant differences of interest (median OS in VTE/AT group 19.26 months, 95% CI 14.50 – 24.08; non-VTE/AT group 16.46 months, 95% CI 1.53 – 31.40, p = 0.35).

Finally, at 9 months (Fig. 1D), the observed trend persisted (median OS in VTE/AT group 23.82 months, 95% CI 18.90 – 28.74; non-VTE/AT group 16.46 months, 95% CI 11.54–21.38, p = 0.50).

## Discussion

The incidence of VTE/AT events in patients receiving ICI has garnered increasing interest in the field of Medical Oncology. Recently, Wang et al. [3] published a review analyzing all available scientific evidence regarding the incidence, risk factors, and management of these events. This review concluded that the higher utilization of ICI has led to a more frequent occurrence of this pathology compared to what is reported in RCTs. This, coupled with the undeniable relevance of cancer-associated thrombosis demonstrated in prospective registries such as TESEO [4], justifies the development of projects like the one presented in this article.

Referencing data from the TESEO registry [4], HNC are not among the oncological entities most frequently associated with VTE/AT. However, a literature search reveals a review article published by Haen et al. [5], concluding that HNCs have biological/molecular characteristics associated with a high risk of VTE/AT, albeit with a low incidence rate. In fact, Monaghan et al. [6] reported a vascular event incidence of 4–5%.

The meta-analysis by Monaghan et al. [6] describes that patients receiving radiotherapy have a higher frequency of vascular events compared to surgery and chemotherapy. Indeed, a recent study [7] concerning surgical procedures reported an incidence rate of 1.3%. However, we found no studies specifically addressing the use of ICI.

In the absence of comparable studies, and considering that all VTE/AT events in our sample occurred in patients receiving nivolumab monotherapy, we decided to review data from the CheckMate 141 study [8], which led to the approval of this ICI modality for patients with recurrent or metastatic squamous-cell carcinoma of the head and neck after platinum chemotherapy. The authors of this manuscript did not report any thrombotic events as secondary toxicity to the treatment. Based on this, it can be inferred that although the thrombogenic risk of HNCs and nivolumab treatment is low, the occurrence of such complications in this patient profile should not be underestimated.

Among our findings, the presence of liver metastases emerged as a predictive factor for VTE/AT in patients receiving ICI. A reasonable doubt arising in this context, necessitating further studies to confirm/disprove this hypothesis, is whether this increased risk is truly attributable to ICI. Previous publications by this group [1, 2] did not reveal this association. However, a recent study argues that liver damage could directly affect platelet activity and increase the risk of VTE/AT [9]. Considering that patients with HNCs tend to abuse alcohol [10], impacting liver health, perhaps ICI is a confounding factor (and therefore its influence on VTE/AT risk is not significant), or perhaps it is a factor that, combined with previous factors, increases thrombotic risk. In any case, further research in this area is warranted.

Before describing the strengths and limitations of this study, it is worth noting that, after a literature review and comprehensive analysis, we have found no justification for the lack of impact on survival in patients who developed VTE/AT compared to those who did not. Our theory is that the low number of thrombotic events recorded (n = 4) may be insufficient to detect a significant impact in survival analysis.

As for the strengths of this study, it is a multicenter project involving centers from different regions of Spain, reinforcing the validity of these results in our healthcare setting. Additionally, it is noteworthy that these data belong to routine clinical practice and are not limited by the inclusion/exclusion criteria of a clinical trial. Therefore, the conclusions drawn can be classified as "real-world data." Lastly, one of the most significant strengths of this study, to our knowledge, is that it is the first series to study a cohort composed exclusively of patients with HNCs.

Despite the described strengths, it is important to acknowledge the limitations of this study. The first is its retrospective nature. However, one limitation stands out, directly related to the recruitment period (2015–2021). The indications for ICI in HNCs are increasing [11], particularly noteworthy is its use in the first line (either in monotherapy or in combination with chemotherapy, the latter of which could increase the risk of VTE/AT due to synergy between the two pharmacological groups in addition to the effect of the underlying oncological disease). This means that if this analysis is repeated in a few years, the percentage of patients receiving ICI in the second line could be considerably lower (which would also affect drug distribution, with an expected increase in the percentage of patients receiving pembrolizumab at the expense of a decrease in the use of nivolumab, as the latter type of ICI is most commonly used in second or later lines of metastatic disease). The last limitation worth mentioning is the size of our sample. Perhaps with a larger sample, a higher proportion of thrombotic events could have been recorded, increasing the likelihood of obtaining statistically significant results.

Despite these observations, it can be concluded that there is still controversy regarding the true risk of VTE/AT associated with ICI in patients with HNCs. Current scientific evidence suggests that the absolute risk of thrombosis is low and that the benefits of ICI treatment outweigh potential risks. However, more prospective studies are needed to assess the risk of VTE/AT in this population and to identify patients at higher risk who may benefit from additional preventive measures, such as antithrombotic prophylaxis.

## Conclusions

Based on our results, we have not observed significant differences in the survival of patients with HNCs receiving ICI who develop VTE/AT compared to those who do not present this complication. However, liver metastases constitute a predictive factor for this event.

## Conflict of interest

The authors have no conflicts of interest related to the development of this research project.

## Data Availability

The authors declare the availability of data analyzed in this study.
